# Resolution of Ultramicroscopy and Field of View Analysis

**DOI:** 10.1371/journal.pone.0005785

**Published:** 2009-06-03

**Authors:** Ulrich Leischner, Walter Zieglgänsberger, Hans-Ulrich Dodt

**Affiliations:** 1 Max Planck Institute of Psychiatry, Munich, Germany; 2 TU München, Institute for Neuroscience, Munich, Germany; 3 Department of Bioelectronics, Institute of Solid State Electronics, TU Vienna, Vienna, Austria; Universidade de Vigo, Spain

## Abstract

In a recent publication we described a microscopical technique called Ultramicroscopy, combined with a histological procedure that makes biological samples transparent. With this combination we can gather three-dimensional image data of large biological samples. Here we present the theoretical analysis of the z-resolution. By analyzing the cross-section of the illuminating sheet of light we derive resolution values according to the Rayleigh-criterion. Next we investigate the resolution adjacent to the focal point of the illumination beam, analyze throughout what extend the illumination beam is of acceptable sharpness and investigate the resolution improvements caused by the objective lens. Finally we conclude with a useful rule for the sampling rates. These findings are of practical importance for researchers working with Ultramicroscopy to decide on adequate sampling rates. They are also necessary to modify deconvolution techniques to gain further image improvements.

## Introduction

**Figure 1:**
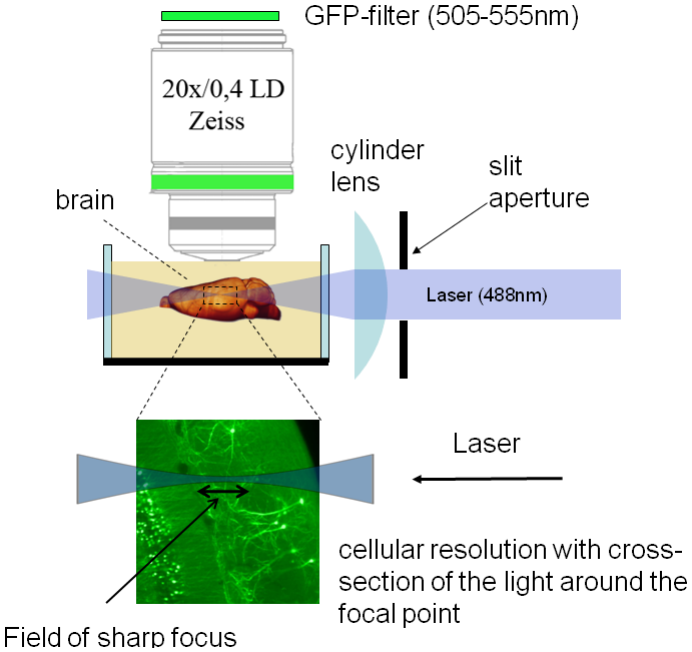
The principle of illumination in Ultramicroscopy: Light (e.g. 488 nm Argon laser) is being focused quoin-like by a cylinder lens. In the area where the beam is focused maximally a quoin-like description is not appropriate and the profile of the illumination beam stays relatively constant. The sample is immersed in the same liquid used to gain transparency. This assures that the illumination beam is not diffracted on the surface of the sample and propagates straight, even inside the sample. By moving the sample, different z-positions can be illuminated and recorded afterwards. In this way we acquired three-dimensional tomographical data.

Ultramicroscopy [1], [2] denotes a microscopical technique in which the sample is illuminated from the side, perpendicular to the direction of observation (fig. 1). We combined this technique with a procedure that makes biological tissue transparent. The basic principle of making biological samples transparent is to replace the water contained in the sample with a liquid of the same refractive index as the proteins and lipids [3]. Although pure proteins, lipids and most other components of a cell are transparent, the inhomogeneous mixture like membranes embedded in water, as in a living cell, is opaque. This opaqueness originates in the differences in the refractive index of bordering components in cells, resulting in scattering of light (Tyndall-effect). By replacing the water contained in the sample with a liquid of the same refractive index of proteins or lipids, scattering effects can be minimized and the transparency of the sample is gained [3]. Spalteholz [3] used a mixture of 1 part benzyl alcohol and two parts benzyl benzoate (abbreviated BABB). This mixture is still a good choice. After this procedure, optical imaging deep inside the biological tissue is possible. With an illumination from the side, layers of the sample can be selectively illuminated and recorded and, thus, tomographical data can be directly acquired. Although this illumination method can be used for scattered light observations as well, we use this method mainly with monochromatic illumination and fluorescence filters recording samples stained with fluorescent markers. This makes this microscopical technique applicable in the research on transgenic-fluorescent animals and allows the use of well established immunohistochemical fluorescence techniques.

As the sample is transparent to a large extent, and immersed in the same liquid used to make it transparent, the illumination beam propagates straight even inside the biological sample and is not interfered by the surface of the sample. For illumination we use an Argon laser (Innova 90, Coherent), for the observation we applied a GFP-filter (505–555 nm wavelength range), recording only fluorescence, and preventing the remaining scattered light from reaching the detector. To perform a three-dimensional recording, all z-positions of the sample were successively illuminated and recorded. This results in a stack of images that can be visualized by computer graphics and are ready for subsequent analysis and image processing.

The practical advantage of this illumination method compared to confocal microscopy is, that the illumination is not done throughout the whole sample in z-direction. By illuminating the focal region from the side, this technique avoids bleaching and intensity losses of the illumination beam by absorption in out-of-focus regions. No fluorescence is generated in the out-of-focus region and therefore has not to be rejected by a pinhole. Hence, with normal lenses we can capture bright images. Additionally, by the sectioning capabilities of the illumination beam, the images have a high contrast. An additional optical advantage is specially given for low magnification objective lenses (about 1×–2.5×). These lenses with large field of view (more than 1 cm^2^) all have a low numerical aperture (NA) and a poor z-resolution in common. With this illumination method only the highlighted parts of the sample contribute to the image and hence to the z-resolution of the 3D data stack. Using objective lenses with a poor z-resolution together with a thin illuminating sheet of light, the z-resolution is mainly determined by the illumination beam. For some combinations the improvement in z-resolution is enormous. In theory, the z-resolution even improves when both resolutions (from the objective lens and the illumination beam) are in the same range, but to a lower extent. The exact relations will be discussed in a later section.

This microscopical technique can image medium-sized samples (1–30 mm) three-dimensionally. It fills the gap between two-photon or confocal microscopy with highest resolution, but smaller field of views (<1 mm^3^), and computer tomography (CT) and magnetic resonance imaging (MRI), that can be used for large objects, e.g humans. An additional advantage is that it is an optical imaging technique that allows visualization of florescent dyes. Well established histological techniques like antibody staining can be used to mark the interesting parts of the sample. Staining can also be done by the design of transgenic animals. In current research sites of altered gene expression in transgenic animals are routinely marked by the additional expression of green fluorescent protein (GFP). This allows the investigation of three-dimensional gene expression in the whole animal. Genetic techniques can also be used to mark specific types of cells, e.g. pyramidal cells in the hippocampus. This can be used for three-dimensional analysis of networks that are formed by these cells. Until now no three-dimensional optical recording technique with micrometer resolution for objects in the size of 1 mm^3^ to 1 dm^3^ existed. In contrast to the above mentioned techniques Ultramicroscopy is very cheap, does not need skilled personal to use, and can be established in any laboratory.

This illumination method was originally named ultramicroscopy [1] and later rediscovered by Voie et al [4] and termed OPFOS (orthogonal plane fluorescence optical sectioning). Later it was termed SPIM (selective plane illumination microscopy) [5]. Although it has already been employed in various research projects [4], [5], [6], we still lack a computation of the resolution with the biological most relevant criterion, the Rayleigh-criterion. This criterion results in an easy formula. Most previous publications approximate the z-resolution by assuming the beam is of Gaussian shape. This leads to incorrect formulae and the given resolution values do not match with our experienced resolution. Additionally, there is no investigation on the sharpness of the illumination beam adjacent to the focal spot. In the theoretical analysis part we present our investigations throughout what size the illumination beam is thin enough to provide an acceptable image, how it widens and how the wider illumination beam affects image quality. This leads to predictions of the size of the field of sharp focus (FoSF, illustrated in fig. 1), describing what field of views can be illuminated with a thin beam. This knowledge helps choosing the right magnification of the objective lens.

There is one publication [7] that does not make a Gaussian beam approximation. Unfortunately they apply the more technical resolution criterion of Full-Width-Half-Maximum (FWHM) of the point spread function (PSF). The advantage of this criterion is that it can be applied to any PSF and describes the resolution of any optical system. The disadvantage is that it is complicated to handle and does not result in an easy formula. In biological research the Rayleigh-criterion is mainly used, because it can be written in an easy formula that allows a fast approximation of the resolution of the optical system. Our investigations here also lead to easy formulae. In contrast to our method the paper that applied the FWHM rule [7] only gives the computed results for some used components. When other components are used it is quite hard to transfer the results. Additionally, the computed values are extremely optimistic and always include an improvement of the resolution by the used objective lens. We think this is misleading. In most cases the objective lens does not improve the resolution when the Rayleigh-criterion is applied. Only theoretically the objective lens improves the resolution in the case when an image reconstruction method in the Fourier-domain is applied. As later discussed this illumination method has mainly advantages because of its sectioning capabilities. It increases contrast and image quality dramatically, but big enhancements of the resolution can not be expected.

Here we apply the two most frequently used approaches to determine the optical resolution in biological imaging: the classical approach using the criterion given by Rayleigh, predicting the subjective resolution of the original raw data, and a more recent approach from signal transmission based on the mathematics of Fourier optics and inverse filtering, predicting the maximum resolution limit that can be reached with ideally working inverse filtering techniques. Inverse filtering is a very important deconvolution technique, but these signal reconstruction techniques were recently refined and extended with other procedures. Deconvolution is now the umbrella term for all these methods that try to reverse a convolution. The theory of inverse filtering, signal recovery from corrupted signal and reachable resolution is much larger [8], especially when prior knowledge of the objects shape is used (e.g. in astronomy) [9], [10]. There are statements that the resolution is only limited by noise, but this requires very advanced image reconstruction techniques. We can not give an introduction of these techniques here and they are not too important in biological imaging. In general biological researchers think that the results of an investigation must be visible in the original data, not only after using sophisticated image reconstruction techniques. In this way deconvolution is a tool to improve the visibility of the results, but it should not be a necessary tool for research. However, mentioning these techniques is important because deconvolution is regularly done to improve biological images. Additionally, with the framework of Fourier-mathematics it is easy to explain the resolution improvement by the objective lens. With these approaches we get an overview on the expected resolution values. Finally we investigate the lateral development of the illumination beam and describe, throughout what size it is of acceptable sharpness and provides good images.

### Mathematical model of mapping biological tissue

**Figure 2:**
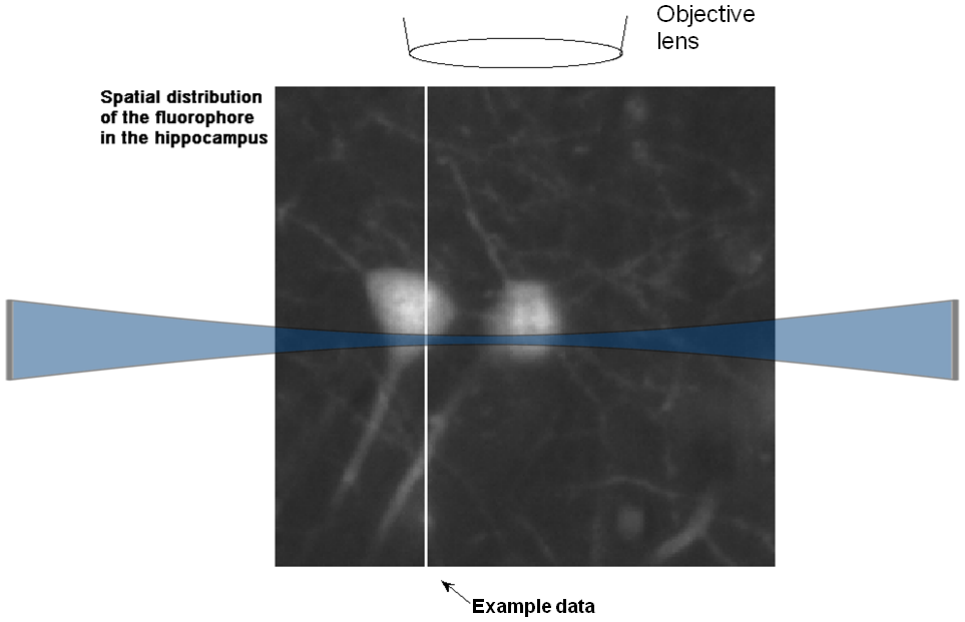
Spatial distribution of the fluorophore. GFP is mainly located inside the neurons in the hippocampus. The spatial distribution of GFP has to be resolved, as demonstrated in fig. 3.

**Figure 3:**
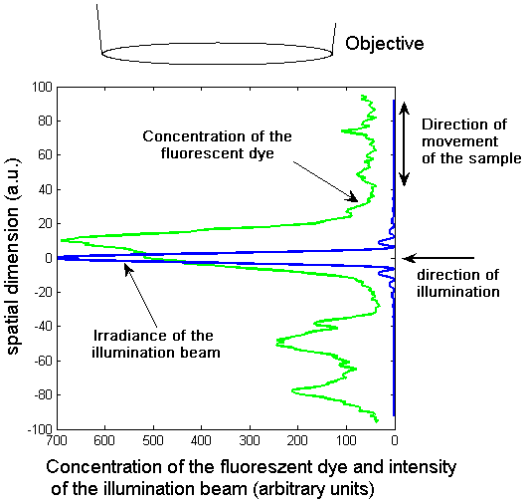
Mathematical model of the mapping principle in one dimension, as shown in fig. 2. The sample is illuminated from the side, and the radiation of the fluorophore is observed from the top. The emission of the fluorescence is directly proportional to the illumination of the fluorophore. As we observe fluorescence from the top, the total detected fluorescence is the integral over the product of the irradiance and the concentration of the fluorophore.

As the lateral resolution of imaging thin slices of biological tissue has been well investigated, we focus on the axial (along the direction of the axis of the objective lens) resolution with this illumination method. In the focal region, only one thin sheet of the sample is illuminated and contributes to the recorded image (fig. 1). To simplify the model we first assume that the depth of focus of the objective lens is far bigger than the contributing cross-section of the light sheet, and ignore bleaching or saturation of the fluorescent dye. We will focus later on the contribution of the objective lens. In this case the axial resolution depends only on the form of the cross-section of the illumination beam. To display the mathematical model we captured data of the concentration of the fluorophore on a line in an axial direction from fig. 2, and plotted it in a mathematical diagram in fig. 3.

We are interested in the spatial distribution of a fluorescent dye, such as GFP, fluorescent antibodies or the background of autofluorescence of the sample. In this model the recorded illumination of a pixel in an image is the integral over the product of two functions, the spatial distribution of the fluorescent dye and the spatial distribution of the irradiance of the illumination beam. When scanning the object, this mathematical model becomes a one-dimensional convolution. Thus, the convolution kernel, i.e. the cross-section of the illumination beam, is essential for predicting the resolution of this illumination method.

## Methods

We used two approaches for calculating the shape of the cross-section of the illumination beam: an approach based on a Fourier transform, and a numerical simulation based on the principle of Huygens.

### Computation by the Fourier method

The Fourier method uses a formula very similar to a Fourier transform. It describes the relationship between the spatial distribution of the electromagnetic field on the back-aperture of the lens and on a plane at focal distance from the lens:
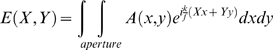
(1)


Herein X and Y denote the spatial coordinates in the focal plane, and x and y denote the coordinates in the aperture plane close to the lens, A(x,y) the distribution of the electro-magnetic field in the aperture plane, *k* the wave number *k* = 2π/λ, λ being the wavelength of the laser light and *f* the focal length of the lens. In the derivation of this equation was assumed that *f* is much greater than the aperture [11]. This formula describes the situation in air. An elaborate derivation of this formula, and information on the factors that were neglected in front of the integral, can be found in [11], [12]. These factors can be regained by normalization. Due to the use of a cylindrical lens, we only performed a unidirectional transformation that additionally simplifies the formula. With these simplifications we can solve the integral as followed:
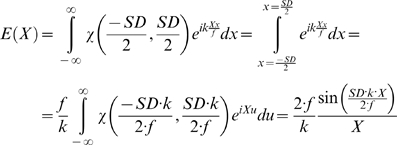
(2)wherein *SD* names the slit diameter and χ the characteristic- (or rectangular-) function ( = 1 inside the interval defined by the boundaries in the brackets, and  = 0 otherwise) representing the slit. For the calculation we assumed a normalized homogeneous illumination. In order to obtain the required irradiance we only have to square equation (2).

### Computation by the principle of Huygens

The Fourier method is only applicable in the focal region. Outside the focal region we computed the cross-section of the illumination beam by applying the principle of Huygens. The principle of Huygens predicates that light propagates as if every point in a wave-front causes a spherical wave, and the resulting wave form is a superposition of these spherical waves. To calculate the illumination of a point at a distance r from the aperture the formula
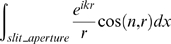
has to be solved. An explanation of this formula can be found in [13]. Here, *n* is the normal vector of the interval of the slit element and *r* the distance from the interval. *cos(n,r)* is the cosine of the angle between r and n. Solving this formula results in the pattern on an projection screen produced by an illuminated aperture, as defined by the integration limits in the formula. We extended this model to calculate beam propagation through lenses by firstly calculating the illumination pattern of a slit on a lens, secondly the pattern on the opposite side of the lens, illuminated by the previously calculated pattern, and finally the distribution of the electro-magnetic field on a projection screen at the desired distance. Because we use a cylindrical lens, we again reduced the model to the one-dimensional case. (The calculation took about one hour for each step on an Athlon 3000+, the properties of the lens like radius, thickness and refractive index were obtained from the database in the program WinLens from Linos). Again we get the required irradiance by squaring absolute values of the final result. (Phase shifts have no influence on the excitation of a fluorophore). This numerical simulation is a relatively easy method that avoids complicated algebraic formulations [14]. Additionally, this method is more precise. We later extend this method to calculate cross-sections through the illumination spot in the direction of light propagation. In this case the analytic formulations become really complicated. We will compare this method with its advantages and differences with the existing analytical methods later. The results of the simulation are given in the next section, together with the results of the measurement of the cross-section of the illumination beam. (The code of the MatLab functions is available upon request).

### The measurement of the cross-section of the illumination beam

**Figure 4:**
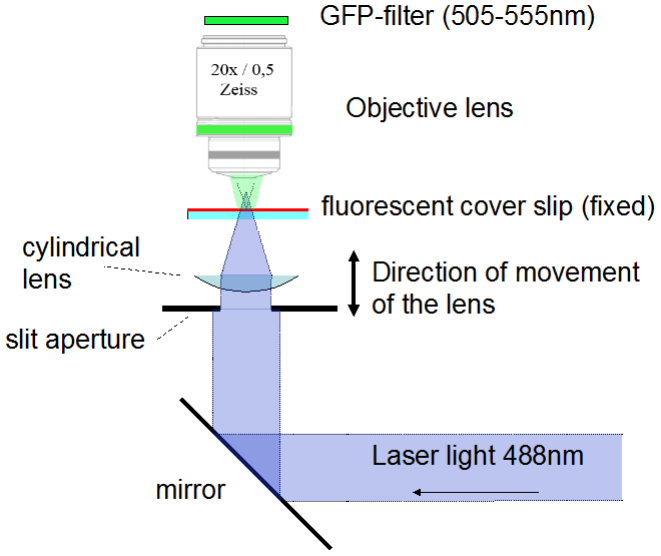
Modified setup for the measurement of the cross-section of the illumination beam. The illumination beam was focused towards the microscope and a fluorescent cover slip was placed in its way. By observing the fluorescence of the cover slip, the cross-sections of the illumination beam was measured. By moving the cylindrical lens, cross-sections of various positions from the focal point can be observed.

**Figure 5:**
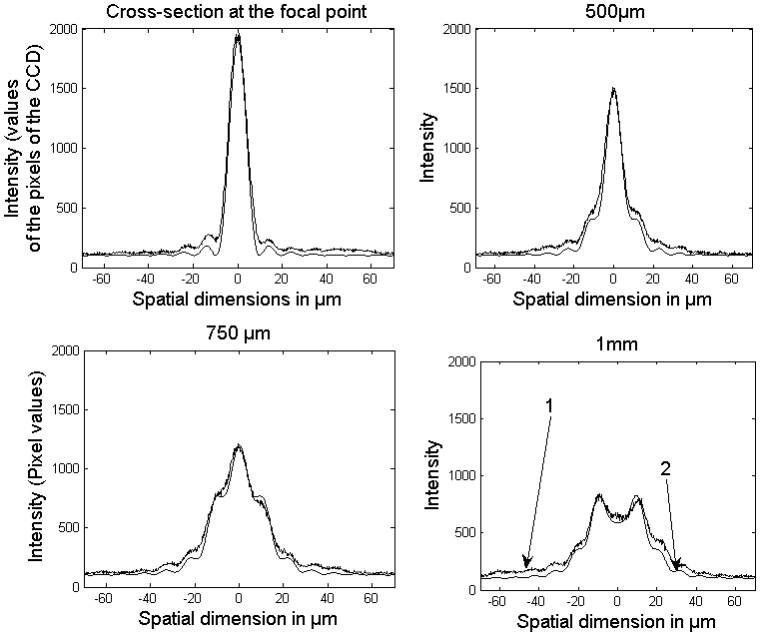
Measured and computed cross-sections of the illumination beam at various distances from the focal point, 1 measured cross-section, 2 numerically computed cross-section (offset and amplitude of the numerical data was fit to the measured data).

The mainly applied technique to measure the resolution of a microscope is simply to record fluorescent beads smaller than the resolution of the microscope. The result is a blurred image of a fluorescent point. By analyzing the image of the blurred point, a prediction of the resolution can be gained. This approach was not possible, because the liquid (BABB) we used to achieve transparency is quite aggressive and dissolved all fluorescent beads we tested. So to measure the cross-section of the illumination beam, we modified the experimental arrangement as shown in fig. 4:

The light (488 nm Argon laser) was directed by a mirror towards the objective lens (Zeiss Plan Neofluar 20×/0.5) and focused by the cylindrical lens and slit aperture. The lens was placed on a micropositioning device and could be moved in a vertical direction. A fluorescent cover slip was placed at a spot around the focal line of the cylinder lens (Molecular probes yellow-green FluoSpheres, polystyrene microspheres (505/515) was dissolved in acetone and the resulting liquid containing the dissolved fluorescent dye was placed and dried on a cover slip. Like this the cover slip was homogeneously coated with the fluorescent dye). The microscope was focused on the cover slip. To record the fluorescence only we applied a GFP-filter (505 nm–555 nm transmission) behind the objective lens. With this arrangement the cross-section of the illumination beam can be measured at any distance from the cylindrical lens. In fig. 5 the results of the measurement of the cross-sections are displayed together with the numerically simulated cross-sections, as described in the previous section.

The computer-simulations yield good predictions for the real shape of the cross-section. Besides the noise, the measured cross-sections are a bit broadened and smeared and not always 100% symmetric. This can be due to the facts that the surface of the lens does not fit completely with the given radius from the catalogue (the accuracy is given in the catalogue), that the slit is not placed exactly on the center of the lens or from an inhomogeneous bleaching of the dye. The positions of secondary minima and maxima are well predicted, and these positions determine the resolution according to the criteria we use here.

### The effect of the immersion medium with a higher refractive index on the cross-section of the illumination beam

**Figure 6:**
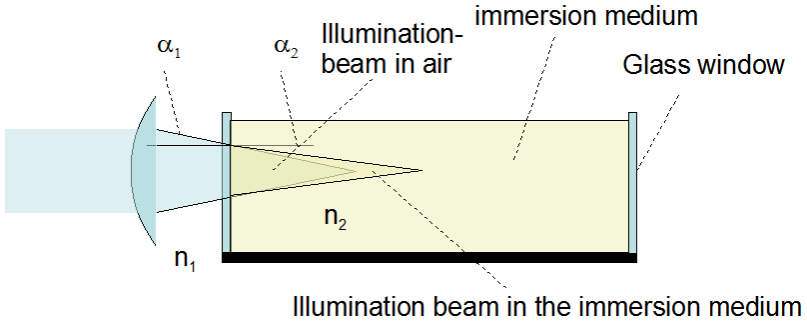
Focusing the illumination beam through a glass window into a different medium with a higher refractive index. The cross-section and thickness stay the same, but the illumination beam is stretched lengthwise by a factor equal to the new refractive index *n*_2_ (deduction in the text).

Some properties of the illumination beam change when it crosses from air through a glass window into the medium with different refractive index (fig. 6). The law of Snellius (*n*_1_sinα_1_ = *n*_2_ sin α_2_, *n*: refractive index) describes the angle change of a ray of light when it enters a medium with a different refractive index. Applying this rule to the bounding rays of the illumination quoin leads to a different focal length: *n*_1_sin(α_1_)≈*n*_1_tan(α_1_) = *n*_1_ *SD*/2*f*_1_ = *n*_2_ *SD*/2 *f*_2_ , so *f*_2_ = *n*_2_ *f*_1_/*n*_1_ . The beam is stretched by a factor equal to the refractive index of the new immersion medium (a value of 1.55 in our case). In contrast the profile of the illumination beam does not change. We can see this by replacing in equation (2) *f*_1_ by *f*_2_ and λ_1_ by λ_2_ = λ_1_/*n*_2_. (In a medium with a higher refractive index is the velocity of light slower, hence the wavelength is shorter. The shorter wavelength compensates the reduced focusing angle.)This relation can be expressed in a short statement: The law of Snellius states that the numerical aperture does not change when the illumination beam is changing the medium. Hence, the resolution does not change as well. This is a useful effect, as a stretched illumination beam laterally enlarges the area of a sharp focused beam, making it more useful for illumination. On the other hand, axial resolution is not increased by the immersion medium.

## Results and Discussion

The theory of resolution, its limits and the applied criteria is enormous [8]. We limit the discussion on the Rayleigh-criterion and to an approach in the frequency domain. The analysis in the Fourier-domain predicts the limits of image reconstruction techniques, mainly known as deconvolution techniques. These two approaches are sufficient for biological researchers who use mainstream techniques in biological imaging. Next we investigate the lateral distribution of a sharp focused illumination beam and the resolution improvements of the used objective lens. Finally we present our sampling rule and explain why we always sampled in this way.

### The Rayleigh criterion

**Figure 7:**
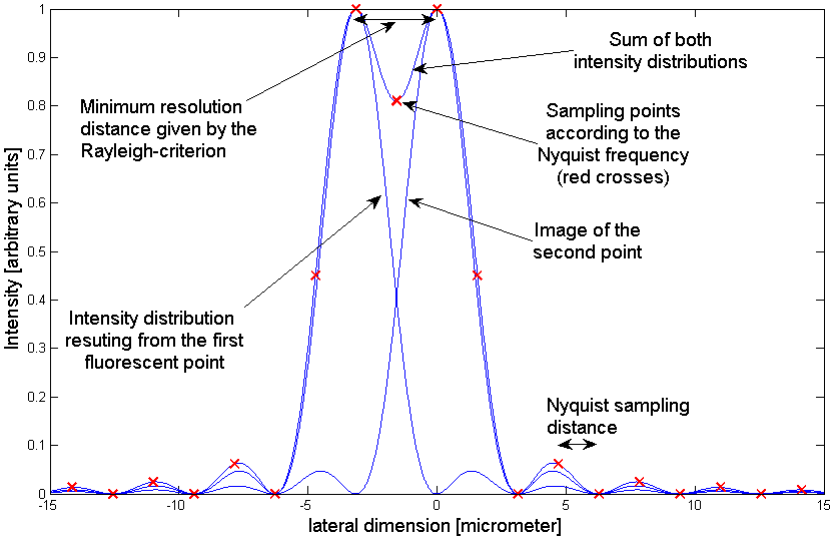
Rayleigh criterion: Two points are recognized as distinct points when they are not closer than the distance between the maximum and the first minimum of the PSF. Red crosses display the Nyquist sampling frequency.

Rayleigh suggested that two points can be subjectively observed as two distinct points when the maxima of their Point Spread Functions (PSF) are not closer than the distance between the first maximum and the first minimum (fig. 7). Applying this to the (sin(x)/x)^2^ function from (2) (*SD k X/2 f = *π), we get a resolution of Δx = *f* *λ/*SD*. (This result is quite close to the known formula *Δx = 0,61 λ/NA* that describes resolution in microscopy for objective lenses. In our case the NA of the illumination beam is defined as *NA = SD/2f* .) In Table 1 we list the components we mainly used together with the resulting resolutions.

**Table 1:** axial resolution calculated by the Rayleigh criterion by the formula Δx = *f* *λ/*SD* and λ = 488 nm.

Used cylinder lenses and slit aperture diameter	Resolution according to the Rayleigh criterion in microns	Useful image size and examined samples
f = 80 mm, SD = 2 mm	19,5 µm	10×10 mm^2^ whole mouse brain, whole mouse embryo
f = 40 mm, SD = 2 mm; f = 80 mm, SD = 4 mm	9,8 µm	3×3 mm^2^ Head of mouse embryo, whole Drosophila
f = 80 mm, SD = 6 mm	6,5 µm	
f = 40 mm ,SD = 4 mm	4,9 µm	0.5×0.5 mm^2^ Section of mouse hippocampus, head of Drosophila
f = 40 mm, SD = 6 mm	3,3 µm	<0.5 mm^2^ High resolution hippocampus, eye of drosophila

Rayleigh-Resolution for different slit apertures and cylinder lenses with illumination light at 488 nm.

### The resolution in Fourier domain

The methods of Fourier analysis we present in this section were mainly developed for radio frequency engineering, but they can be transferred to two- or three-dimensional image signals as well. It is a known phenomenon in the transmission of radio signals that the attenuation of a signal is dependent on the signals frequency. Some frequencies can be transmitted through air very easily, while other frequencies are more attenuated. The same is true for other components like amplifiers, cables and antennae. Methods of Fourier analysis are very useful to predict the change of the shape of a complex signals that consists of several frequencies. To compute the shape of a signal after it passed e.g. a cable you first need to know how much a specific frequency is attenuated by the cable. Next, by a Fourier transformation the original signal is resolved into its frequency components and the specific frequency components are attenuated according to the cables properties. After an inverse Fourier transformation of the modified frequency components, we get a good prediction how the signal looks after it passed the cable.

This approach can be transferred to three-dimensional image signals. Very helpful is the fact that mapping the sample is based on a convolution, hence the convolution theorem can be applied. This theorem states that the convolution kernel determines how much a specific frequency is attenuated. In noise-free signals this attenuation can be reversed by simply Fourier-transforming the recorded signal, amplifying the specific components according to its attenuation factor and finally a reverse Fourier-transformation of the modified frequencies. This procedure is known as “inverse filtering” or “deconvolution”.

The limits of this procedure are first of all noise. The second and more general limit is determined by the convolution kernel. Some frequencies are simply not transferred and are completely erased in the recorded signal. These frequencies can not be recovered by an inverse filtering procedure.

The convolution theorem states that the Fourier transformation of the convolution kernel predicts how much a specific frequency is attenuated. The convolution kernel is in our case the cross-section of the illumination beam. There is a very elegant way of computing the Fourier transformation of this cross-section. Because the cross-section of the illumination beam is the square of the Fourier-transformation of the slit function, as shown in function (2), there is very little to compute. Because a multiplication of two functions corresponds to a convolution in the Fourier domain, the square can be written as a convolution of the slit function with itself. 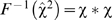. In a long form the function is
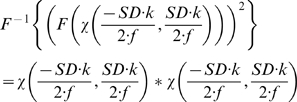
(3)


We can solve a convolution of two rectangular functions analytically. The result is a triangular function with twice the width of the rectangular function from (2). This function is also known as Optical Transfer Function (OTF), as it describes with what amplitude a specific frequency is being transferred through the imaging system. In the figure showing the Optical Transfer Functions graph 1 displays the best focused OTF. (We are lucky that all functions are symmetric, hence the Fourier-transforms are real with no imaginary components. Imaginary parts in the OTF would describe phase-shifts of a specific frequency, but as they don't appear we ignore that part of the theory)

There is a cut-off frequency, defined by *DS 2 π/(λ f)*. Higher lateral (angular) frequencies in axial direction cannot be transferred by this illumination and are completely deleted by this mapping method.

To interpret these values you have to consider that the Fourier-tranform as written in formula (2) does not transform into the normal frequency space, but in the angular frequency space. A sine-wave with a frequency of 1 per meter would result in a frequency of the value 2π/meter with the upper formula. In normal frequency space a frequency is defined by 1/(wave-length), while in angular frequency space it is defined by 2π/(wave length). We have to correct these values to normal frequencies by dividing by 2π and come to *SD/λ f*. The wave-length of this cut-off frequency is identical with the Abbe-limit *(λ/2 NA)*, which was developed to describe the resolution limit of spherical lenses. The wave-length of this cut-off frequency is the same like applying the Rayleigh-criterion on the PSF.

### The Nyquist frequency

A practical question for researchers in biological imaging is how many pixels are needed in-between two objects that these objects are clearly separated. Researchers normally cite a theorem that is known by the names Nyquist, Shannon, Whittaker (and sometimes Kotelnikow) and all combinations of these four names. The theorem proposes a pixel size that is useful in practice.

The theorem answers the questions, how a function can be completely reconstructed when we only know the values of the function at single points (the sampling points). The answer is: In case that the function is band-limited and the points are equally spaced, the distance between the points must be at least half of the wavelength of the maximum frequency present in the signal. This frequency of the sampling points is known by the name Nyquist-frequency. In fig. 7 we show how the points must be spaced when the distance of the Rayleigh-criterion is seen as the wavelength of the limiting frequency.

In biological imaging we do not sample from single points, but integrate the function over a certain distance. In this case the results of the sampling distances are identical. The big advantage of this sampling rule is, that it is easy and clear and it matches well with practical requirements.

### The resolution around the focal area

**Figure 8:**
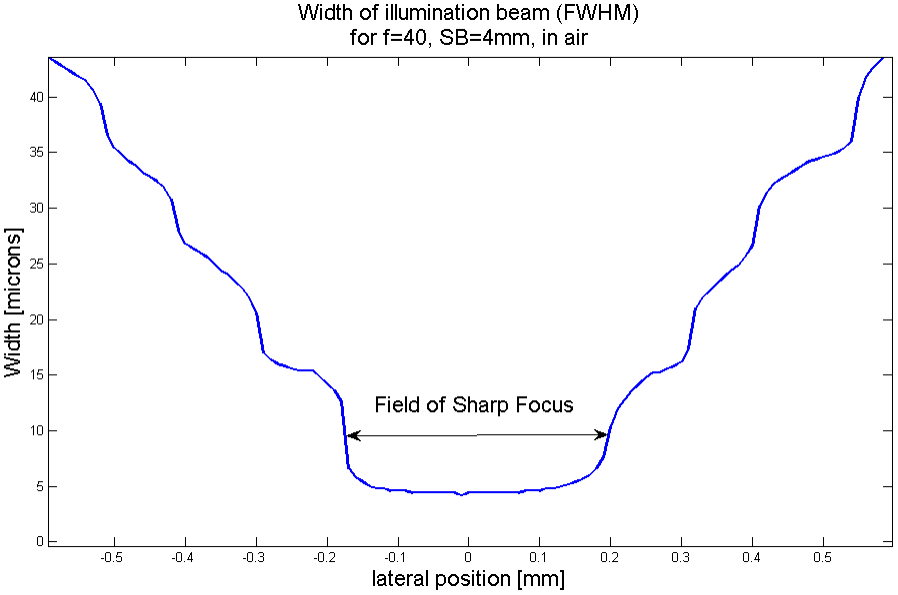
Thickness (FWHM) of the illumination beam versus distance from the focal spot.

Besides the focal spot of the illumination beam we cannot apply the Rayleigh-criterion, because there is not always a first minimum or the secondary maxima are larger than the first maximum (fig. 5). In this case we measured the FWHM of the illumination beam of the computationally simulated cross-sections. At the focal spot the FWHM-criterion predicts a resolution of 4.2 µm, quite close to the 4.9 µm predicted by the Rayleigh-criterion. In fig. 8 we plot the value of the FWHM of the cross-section of the illumination beam versus the lateral position from the focal spot.

The thickness of the illumination beam is varying stepwise. This is caused by the fact that we mainly count the number of secondary maxima that contribute to the FWHM-distance. We state here that a beam is well focused as long as only the first maximum contributes to the FWHM-value, no secondary maxima. This is in agreement with our subjective impression of image quality and with the observation, that the image quality is relatively constant in the center of the image, but then decreases rapidly.

To investigate the depth-resolution of a cylindrical lens we can use the results for normal objective lens systems. The depth-resolution there according to the Rayleigh-criterion is given by *2nλ/(NA)^2^* [15], [16]. In our case the numerical aperture is defined by *SD/2f*. With the previous described functions we simulated several beam distributions around the focal spot with different components and cylindrical lenses. We found, that a function with a quadratic relationship between the numerical aperture and the field of sharp focus describes the simulations for cylindrical lenses quite well. Because the field of sharp focus, as defined above, does not match with a Rayleigh-resolution, we have to find an additional coefficient. All these relationships result in the following formula:
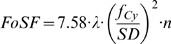
(4)


Here *FoSF* denotes the field of sharp focus, *f_Cy_* the focal length of the cylinder lens, *SD* the width of the slit diameter, *n* the refractive index of the immersion medium and λ the wavelength of the illuminating light. The result is given in the unit of λ (normally nm).

In biology very often the size of the specimen is given, e.g. a head of drosophila. This size defines the field of sharp focus, that next predefines the axial resolution. We can unify the formula for the field of sharp focus (4) with the one for the axial resolution (Δx = *f* *λ/*SD*):
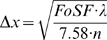
(5)


**Figure 9:**
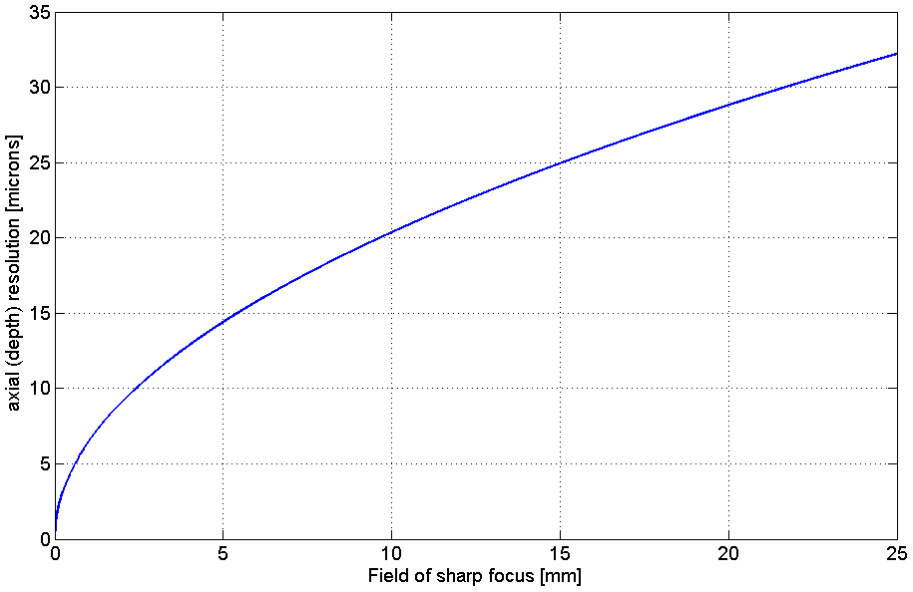
If the necessary size of the field of view is given (e.g. the head of a drosophila), the thickness of the illumination beam must be constant throughout that size. This predefines the depth-resolution. Because we have a square-root relationship, highest depth-resolutions (<5 microns) are difficult to reach and are accompanied with and extreme small field of sharp focus.

This formula describes the axial resolution (in the standard Rayleigh-case) for a given specimen size obtained with the optimal selection of slit diameter. Practical examples are given in table 1 and fig. 9.

Very often our images cover larger field of views than the given values from equation (4). Like this only the center of the image is illuminated with maximum sharpness. The worse resolution on the sides of the images is acceptable and does not disturb the image severely. To record large objects, we used a trick. We recorded each image twice, once illuminated from one side and the other illuminated from the opposite side. For this recording procedure we adjusted the illumination beams in a way that one illumination beam provides a sharp image on one side of the sample and the other illumination beam on the other side. We then merged the two recorded images to one final image that only consists of sharp parts of the original images[17], [18]. In this way we increased the field of sharp focus and the image quality.

**Figure 10:**
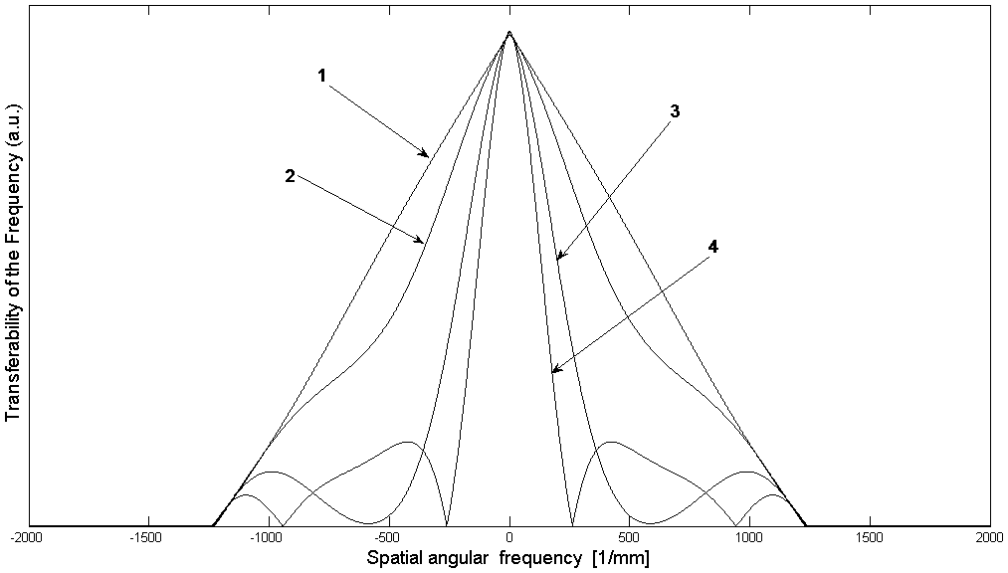
Amplitudes of the axial Optical Transfer Function at various distances besides the focus region, focused by a cylindrical lens (f = 40 mm) and 4 mm slit width, 1 at the focal point, 2 at 0,1 mm distance, 3 at 0,2 mm distance, 4 at 0,3 mm distance from the focal point.

In Fourier-optics, the relationship between the predicted resolution and the distance from the focal spot is different. Since there the OTFs predicates the extent of information on a lateral frequency that is being transferred by the measurement, we computed the Fourier-transformed cross-sections of the simulated illumination beam from various distances besides the focal spot. The results are given in fig. 10.

Higher lateral frequencies are less transferred by a less focused beam. Surprisingly the cut-off frequency does not alter, hence with perfectly working deconvolution techniques all the information could still be recovered, even with a badly focused beam. This theoretical statement has no practical implication, since noise is always present in images and prevents the recovery of weakly transferred lateral frequencies. Statements on the resolution in Fourier-optics need to consider the noise level. The spectral components of white noise, the standard noise model, have the same amplitudes all over the spectrum. This value can serve as a threshold level. Lateral frequencies that exceed this threshold level can be regarded as resolvable. Without knowledge of the noise level we cannot say, how weak a signal can be that it can still be recovered.

In fig. 10 we plot the absolute values. The values could be negative. A negative frequency would correspond to a phase shifted frequency with a shift of 180°. It is important to consider this in the implementation of inverse filtering algorithms.

### The combined resolution of the objective lens and the illumination beam

The PSF of the objective lens in non-coherent imaging is given by the intensity distribution of a beam focused by the objective lens [19]. In the case when illumination and detection can be separated, both PSFs must be multiplied. In confocal imaging for example, the illumination and detection is done by the same lens, hence in a first approximation the PSF can be squared [19]. (The wavelength of excitation and emission are not identical, hence the PSFs are also not identical. Squaring is not exactly right, but it is a good first approximation). In order to derive resolution values of the combined optics it is essential to know the shape of the focal spot of the objective lens.

**Figure 11:**
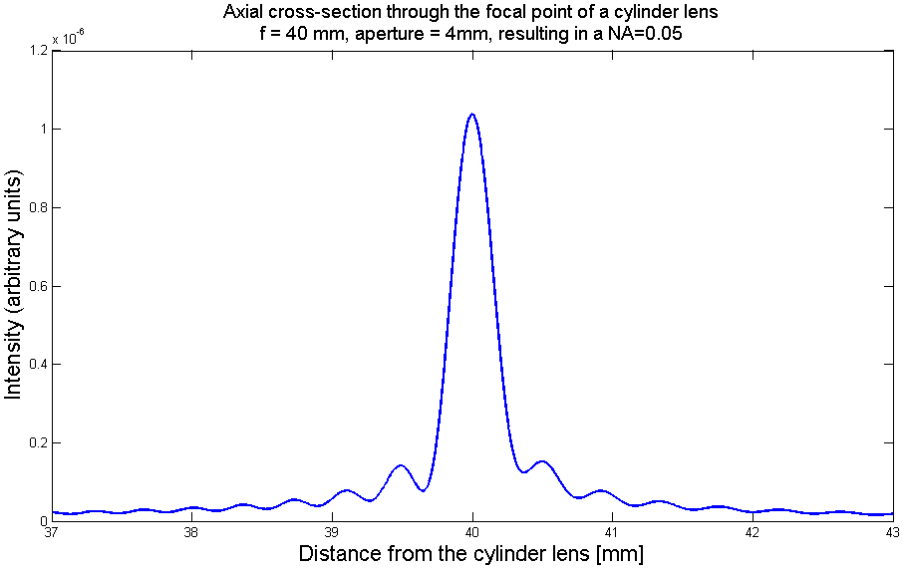
Axial cross-section of an illumination beam of a cylinder lens with low NA. The shape is similar to a cross-section through the focus spot of an objective lens. The location of secondary maxima and minima are only determined by the NA, so this can be seen as the axial component of the PSF of the objective lens. As objective lens systems normally have several corrections (chromatic-, spherical-, and plancorrection), this function can only be used for qualitative discussions.

An axial cross-section through such a focus spot of an objective lens looks very similar to a cross-section of our illumination beam (fig. 11). It consists of a main maximum surrounded by several maxima of higher order. The algebraic calculations for their computation are complicated and non-uniform for low NAs and high NAs [15], [16]. For low NA the distance between the first minimum and the main maximum is given by *2nλ/(NA)^2^* , for high NA the value is a bit smaller. We did not often use objective lenses with NAs above 0.4, so we neglect the case of a high NA. (High numerical apertures generally do not allow large working distances. Large working distances are necessary for three-dimensional imaging, the main strength of this technique). For a discussion we simulated an axial cross-section through an illumination beam with the numerical methods presented earlier and plotted it in fig. 11.

It is surprising that the cross-section is not symmetric and the minima do not reach zero, as stated in previous mathematical formulations [15], [16]. Additionally they also exhibit a non-symmetric appearance with less pronounced minima behind the focal spot. We also saw this asymmetry in our measurements, hence this method is more precise. Spherical aberrations would result in a similar asymmetry of the cross-section. Another reason for this asymmetry could be that the slit aperture is not located in the Fourier plane, but in some distance from it.

**Figure 12:**
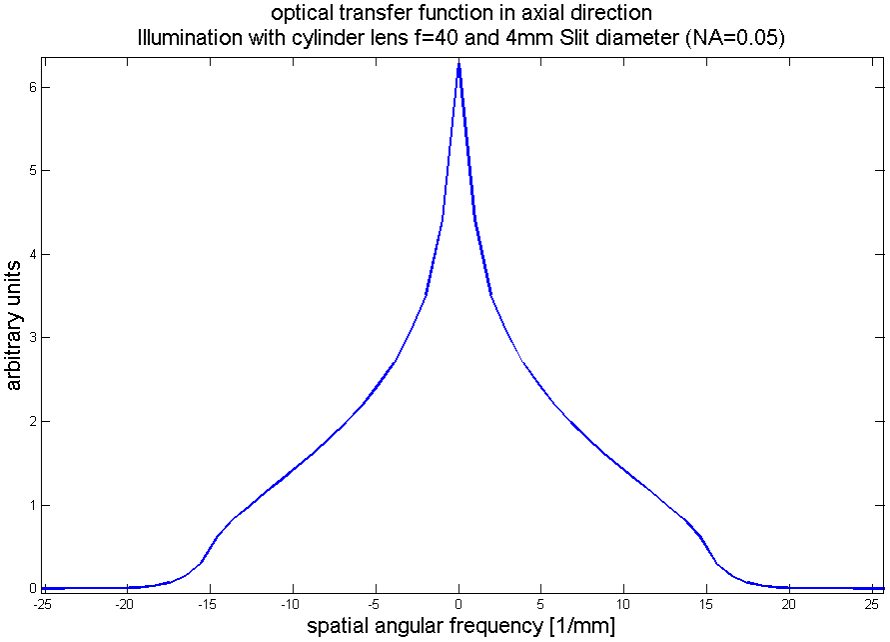
Model of the axial component of the OTF of the objective lens with a NA of 0.05. As the two PSFs look similar, its Fourier-transforms are similar as well, as can be compared to fig. 10.

This function can be used as a model for the detection PSF of an objective lens with low NA. (By using an ordinary lens instead of a cylinder lens the main maximum may be more pronounced, but the location of secondary maxima and minima are the same, since they are determined by the NA (defined for a cylinder lens with a slit aperture as *SD/2 f* ) The value *2nλ/(NA)^2^* predicts the resolution according to the Rayleigh-criterion. Multiplying two functions like in fig. 11 and fig. 5 results in a function of similar shape. The distance from the main maximum to the first minimum is predefined by the smaller distance of both functions. Hence, according to the resolution criterion of Rayleigh the better resolution value defines the overall resolution. In the theory of inverse filtering the situation is different: A multiplication corresponds to a convolution in the frequency domain, hence the convolution of the two OTFs has to be computed. In fig. 12 we plot the amplitude of the Fourier-transform of the function displayed in fig. 11.

As the two PSFs look similar, the transfer functions are similar as well. There is a cut-off frequency as well, again at a frequency of 1/(Abbe-Resolution), (real frequency, not angular frequency). A convolution of two functions with finite support leads to a function where the support of both functions is added. Hence, the cut-off frequencies can be added and a resolution improvement can be stated. This is again the theoretical limit, only the case when an inverse filtering procedure is applied and far from practical values. A formula that gives a more realistic description of the resolution improvement is given by:
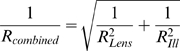
(6)


Its derivation is as follows: In statistics, the probability distributions of the sum of two independent random variables can be modeled as a convolution. Variances of independent random variables can be added. As the standard deviation (the square root of the variance) is the interesting value, we come to this formula. Maximally the resolution can be improved by a factor of 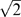 in the case when both resolution values are the same. When both resolution values are not in the same range, the better one dominates the overall resolution. The value of 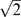 was first proposed in [20].

### Light sheet thickness and image quality

The resolution of an optical system is normally measured by simple test object like small fluorescent beads, ronchi rulings or test targets, and the theory makes a good description of this simplified situation. These test objects are normally very bright, with sharp edges and the pattern on it are isolated not to interfere with each other. Especially the last point is very different in real biological samples. Interesting structures are very close, and the challenge is to resolve one location with high resolution and suppress interferences from nearby areas. Image quality is much better when unsharp objects from the background are removed. Contrast is the name of the image quality feature that describes the amount of background present in the image. It is defined by the ratio of the signal amplitude (peak-to-peak) to the overall amplitude including the background. The subjective image quality is highest when the contrast is highest. Restricting the discussion on the resolution only to the mathematical criteria is a bit misleading, because it would ignore the contrast and image quality.

Because in theory the objective lens itself could already provide the good z-resolution, we made a test. We recorded a z-stack of a drosophila sample with epifluorescence illumination and gave the data to our contact person of a big microscope vendor and asked him to make a deconvolution with their software packages. In epifluorescence illumination the sample is illuminated from above through the objective lens. In theory deconvolution packages should be able to remove out-of-focus blur and reconstruct the in-focus section, but the result showed the opposite. We could hardly recognize that we recorded a drosophila. Epifluorescence illumination generated a lot of out-of-focus fluorescence that results in a high background. The out-of-focus blur is much brighter than the fluorescence of the sharp in-focus area. The images are unclear and blurry, or in technical terms: The contrast is very low. This experiment just showed that epifluorescence illumination is not a suited technique for three-dimensional recordings, although in theory the depth resolution could be the same. We need a powerful technique to reject the background, otherwise the image quality is too bad to reconstruct the three-dimensional structure.

The contrast and hence the image quality is best, when no fluorescence is generated in out-of focus regions. This is the case when the light sheet is thinner than the depth-resolution of the objective lens. So we have two statements: The better resolution defines the overall resolution, and (because of image quality reasons) the light sheet resolution has to be better than the objective lens depth resolution. Hence the light sheet determines the overall resolution (with the presupposition that image quality is optimized. If you disregard image quality you could argue for better resolution values.) This describes the situation when we have a lot of background fluorescence. In the case when you image sparsely distributed fluorescent beads in a transparent liquid there is almost no background and the objective lens itself provides a good depth resolution. To image biological samples we always selected components in a way that image quality is optimized. With the use of deconvolution methods you could expect some improvements by the objective lens (we argue for a factor of 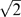), but this is difficult to get. First of all because this illumination technique is a bit exotic and it is not included as an option in standard deconvolution packages. Secondly, the overall PSF is more variable, because in practice the focal plane of the objective lens and the center of the light sheet are not perfectly aligned. This is the case when the microscope is not perfectly focused on the illumination beam. This additional parameter complicates the prediction of the real PSF and limits the efficiency of the algorithms. Imaging is much easier when the depth-resolution of the objective lens is much larger than the light sheet, because then an optimum alignment is not so important, but in this case there is not a big improvement in the depth-resolution by the objective lens. So altogether, the depth-resolution is mainly determined by the light sheet thickness. Possible resolution improvements by the objective lens are not dramatic and should not be overrated.

We generally used objective lenses with axial resolution worse than the resolution of the illumination beam, but close to the resolution. If we had the choice between two objective lenses, we took the one with the higher NA. This has the advantage of a better x-y resolution and, as more light is being caught from the sample, the illumination time can be reduced. This avoids fading and reduces the noise in the images.

### A practical rule for the sampling rates

For an easy rule we propose to sample the object in the same z distance as the spatial size of pixels in x-y direction of the image. This results in voxels with a cubic shape. Cubic voxels are very useful for displaying the data with computer graphics. It avoids previous resampling. Best results, e.g. the images and films from [2], were obtained by sampling cubic voxels. Computer graphics cannot handle other data sizes easily. When this rule was not followed (in the case when we sampled according to the Rayleigh-resolution) the visualization normally looked very poor, despite the high quality of the images. (For best results we used volume-rendering techniques to display the data. When the voxels are not cubic, the newest graphic cards already perform an interpolation to fill up the gap between two adjacent voxels. This interpolation is only done in the direction of the frontal plane of the object. When we create a movie with a rotating sample, the front plane of the sample changes, and therefore the direction of interpolation also changes. This sudden change in the visualization appears unnatural. With cubic voxels this effect does not appear. This effect will only be visible in high quality images with sharp objects. In blurred and unsharp images it will not be visible.) Cubic voxels are also very useful when we apply image reconstruction techniques, where the dataset has to be rotated. Multiple recordings of the same sample rotated by different angles and subsequent composing of the final data of the best parts of each data set returned images with a good constant overall quality [17], [18], [21]. As the axial resolution is about 5 to 10 times worse than the x-y-resolution (depending on the used lenses), a promising approach is to get two recordings of the same sample rotated by 90° and extract the location of the fluorophore from the data set where they are mapped sharply [17], [22]. Rotations and the subsequent merging of data sets are easier to perform with cubic voxels. The sampling distance then depends more on the used components like pixel size on the CCD detector, the used diminution lens before the detector chip and the magnification of the objective lens. We mainly used standard components with an average pixel size of the CCD (a CoolSnap cf^2^ with a pixelsize of 4.65×4.65 micrometer).

To oversample the object along z from the Rayleigh-resolution value is justified by several reasons: First, having more data points can be used to reduce noise. This will result in a better image quality. Second the objective lens contributes to an improvement by a factor of about 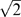. Third, by applying deconvolution techniques the image quality additionally improves. The object is oversampled by a factor of about 3.This is a good compromise. As long as bleaching is not a problem, it does not degrade image quality. Sampling according to this resolution rule results in images that can be immediately displayed with computer graphics, an extremely practical feature.

The disadvantage of this sampling rule is that more computer memory is needed. An average data stack (1400×1000 pixels×700 images) had about 2 GB raw data. Computer memory and space on the hard disk were never an issue. The memory of the graphics card is the limiting factor. There the data is displayed in an 8 bit format, with half the size that is needed for storage. With a graphics card with 1 GB memory we were able to display the data in most cases without previous downsampling.

### Further prospects

The rule for the sampling distances is of practical importance for researchers working with this method. By following these rules, oversampling and undersampling can be avoided. If later image quality should be additionally increased by an image reconstruction technique like a deconvolution, the proposed sampling rates still offer enough data points that an improvement is possible.

Next we investigate the size of the field of sharp focus. For a given specimen size the investigator can calculate the suitable slit diameter and then he will be able to predict the axial resolution. This is important for the planning of experiments.

The focus of this paper is on the investigation of the cross-section of the illumination beam, that is the axial PSF of the illumination. A precise knowledge of it is essential for modifying deconvolution algorithms for ultramicroscopy. Standard image processing packages (e.g. ImageJ) do not include this deconvolution option yet. With this paper a computer scientist with no deeper knowledge in optics is able to model the PSF and extend the functional range of these free software [23] packages.

A promising approach that works already without modified deconvolution algorithms is to use a PSF from the illumination beam that looks similar to the PSF from the objective lens. This can be achieved by using a slit with a width of *SD = f NA^2^λ_exitation_/2nλ_emission_*. In this function *NA* denotes the numerical aperture of the objective lens. In this case the (one-dimensional) illumination PSF and the detection PSF are very close to the confocal case, hence deconvolution algorithms developed for confocal imaging can be applied. We already reported in [2] that we obtained good results when deconvolution algorithms were used in this way.
